# Knockdown of Heparanase Suppresses Invasion of Human Trophoblasts by Activating p38 MAPK Signaling Pathway

**DOI:** 10.1155/2018/7413027

**Published:** 2018-04-17

**Authors:** Guanglu Che, Yanyun Wang, Bin Zhou, Linbo Gao, Tao Wang, Fang Yuan, Lin Zhang

**Affiliations:** ^1^Laboratory of Molecular Translational Medicine, Center for Translational Medicine, Key Laboratory of Birth Defects and Related Diseases of Women and Children (Sichuan University), Ministry of Education, Department of Obstetrics and Gynecology, West China Second University Hospital, Sichuan University, Chengdu, Sichuan 610041, China; ^2^Department of Immunology, West China School of Preclinical & Forensic Medicine, Sichuan University, Chengdu, Sichuan 610041, China; ^3^Center of Reproductive Medicine, Department of Obstetrics and Gynecology, West China Second University Hospital, Sichuan University, Chengdu, Sichuan 610041, China

## Abstract

Preeclampsia is a pregnancy-related disease with increasing maternal and perinatal morbidity and mortality worldwide. Defective trophoblast invasion is considered to be a major factor in the pathophysiological mechanism of preeclampsia. Heparanase, the only endo-*β*-glucuronidase in mammalian cells, has been shown to be abnormally expressed in the placenta of preeclampsia patients in our previous study. The biological role and potential mechanism of heparanase in trophoblasts remain unclear. In the present study, stably transfected HTR8/SVneo cell lines with heparanase overexpression or knockdown were constructed. The effect of heparanase on cellular proliferation, apoptosis, invasion, tube formation, and potential pathways in trophoblasts was explored. Our results showed that overexpression of heparanase promoted proliferation and invasion. Knockdown of heparanase suppressed proliferation, invasion, and tube formation but induced apoptosis. These findings reveal that downregulation of heparanase may contribute to defective placentation and plays a crucial role in the pathogenesis of preeclampsia. Furthermore, increased activation of p38 MAPK in heparanase-knockdown HTR8/SVneo cell was shown by MAPK pathway phosphorylation array and Western blotting assay. After pretreatment with 3 specific p38 MAPK inhibitors (BMS582949, SB203580, or BIRB796), inadequate invasion in heparanase-knockdown HTR8/SVneo cell was rescued. That indicates that knockdown of heparanase decreases HTR8/SVneo cell invasion through excessive activation of the p38 MAPK signaling pathway. Our study suggests that heparanase can be a potential predictive biomarker for preeclampsia at an early stage of pregnancy and represents a promising therapeutic target for the treatment of preeclampsia.

## 1. Introduction

Preeclampsia (PE) is a pregnancy-related disease that is accompanied with new-onset hypertension and multiple organ dysfunction; this disease occurs after the 20th week of gestation [1]. PE affects approximately 5–8% of pregnancies and is the leading cause of increasing maternal and perinatal morbidity and mortality worldwide [2]. As the etiology and pathogenesis of PE remain unclear, a predictive method for the disorder is still lacking. Placental functional deficiency is considered a critical factor for the occurrence and development of PE [3, 4]. During early pregnancy, several extracellular matrix (ECM) degradation-related proteins are expressed by extravillous trophoblasts (EVTs) to ensure normal trophoblast invasion [5]. The ECM is an integral component of the stroma and plays an important role in regulating cell growth and differentiation, tumor cell invasion, and metastasis [6]. When enzymatic degradation of the ECM is insufficient to allow EVT invasion of the myometrium, impaired invasion could lead to defective placental implantation and remodeling of the uterine spiral artery [4]. Consequently, substantial circulating inflammatory factors are released by the placenta leading to an excessive systemic inflammatory response and blood vessel dysfunction.

Heparanase (HPSE) is the only endo-*β*-glucuronidase in mammalian cells that selectively cleaves the heparan sulfate (HS) side chains of heparan sulfate proteoglycans (HSPGs), an important component for the remodeling of basement membranes and ECMs [6, 7]. It has been demonstrated that the expression and localization of HPSE are associated with the regulation of cell proliferation, vascular remodeling, and vascular stability by accommodating HSPG degradation and recombination [8]. High levels of HPSE have been detected in many malignancies and tissues, where it is involved in tumor cell growth, angiogenesis, tumor invasion, and metastasis by degrading and remodeling the ECM [6, 9, 10]. We have previously demonstrated that the expression and localization of HPSE were abnormal in the placenta of PE patients [11]. However, the function of HPSE in mediating the biological behavior of EVT in vitro and the underlying mechanisms remain unexplored.

In the present study, we demonstrated that HPSE had a considerable role in the biological behavior of EVT. Overexpression of HPSE promoted EVT growth and invasion. Knockdown of HPSE was associated with the suppression of EVT proliferation, invasion, and tube formation but an acceleration of apoptosis. Furthermore, we discovered that downregulation of HPSE attenuated the invasion of EVT by activating the p38 MAPK pathway.

## 2. Materials and Methods

### 2.1. Cell Line Authentication

The human first-trimester EVT cell line HTR8/SVneo cells were cultured in RPMI 1640 growth medium (HyClone, USA) supplemented with 10% fetal bovine serum at 37°C with 5% CO_2_. The genomic DNA of HTR8/SVneo cell was isolated with a Tissue/Cell Genomic DNA Extraction kit (BioTeke, China) according to the manufacturer's protocol. The short tandem repeat (STR) DNA profiling of HTR8/SVneo was analyzed with an AGCU EX22 fluorescent detection kit (AGCU, China) which can coamplify 22 STR loci (CSF1PO, FGA, TH01, TPOX, vWA, D3S1358, D5S818, D7S820, D8S1179, D13S317, D16S539, D18S51, D21S11, D2S1338, D6S1043, D12S391, D19S433, D2S441, D10S1248, PentaD, PentaE, and Amelogenin) with an ABI 3130 Genetic Analyzer (ABI, USA) according to the manufacturer's manual.

### 2.2. Construction of Stably Transfected Cell Lines

Short hairpin RNA (shRNA) that targets HPSE (CCTTTGCAGCTGGCTTTAT) was constructed and cloned into the *pLKD-CMV-G&PR-U6* vector. The open reading frame (ORF) of the HPSE gene was cloned into the *pLenti-EF1a-EGFP-P2A- Puro-CMV* vector. The sequences of primers for HPSE gene synthesis were as follows: forward 5′-TGAACCGTCAGATCGAATTCGCCACCATGCTGCGCGCTCGAAG-3′ and reverse 5′-TCATCCTTGTAGTCGAATTCGATGCAAGCAGCAACTTTGG-3′. Overexpression and knockdown vectors contained GFP reporter genes to detect transfection efficiency. The puromycin resistance gene was used as a selection marker for the stringent phenotypic selection of stably transfected HTR8/SVneo cells in the presence of 2 *μ*g/ml puromycin. The stable clones were maintained in 1 *μ*g/ml puromycin.

### 2.3. RNA Isolation and qRT-PCR

Total RNA was extracted from cells using the TRIzol reagent (Invitrogen, USA) according to the manufacturer's instructions and quantified by spectrophotometry (Nanodrop 2000, USA). cDNA was synthesized from 1 *μ*g of total RNA using an iScript™ cDNA Synthesis Kit (BioRad, USA). Real-time PCR was performed using SsoFast™ EvaGreen® Supermix (BioRad, USA). The sequences of PCR primers were as follows: *β*-actin forward 5′-GTCATTCCAAATATGAGATGCGT-3′ and *β*-actin reverse 5′-GCTATCACCTCCCCTGTGTG-3′ and HPSE forward 5′-CCTGAAGGCTGGTGG AGAAG-3′ and HPSE reverse 5′-GGTAGCAGTCCGTCCATTCA-3′. HPSE mRNA levels were evaluated by the relative CT method (2^−ΔΔCt^ method), and the results were normalized to *β*-actin.

### 2.4. Cell Invasion Assay

Cell invasion assays were performed using Corning chambers with transwell filters (8 *μ*m pore size, 24 wells, Corning, UK). The chambers were coated with 25 *μ*l of diluted Matrigel matrix (Corning, UK). 5 × 10^4^ cells were counted using a Neubauer hemocytometer, suspended in 100 *μ*l of RPMI-1640 medium (FBS-free), and seeded into the upper chamber. RPMI-1640 medium (600 *μ*l) supplemented with 10% FBS was added to the lower chamber. After 24 h of incubation, the cells were fixed using methanol and colored with 0.2% crystal violet. The number of colored cells was counted using an inverted microscope in 4 random fields (Olympus, USA).

### 2.5. Tube Formation Assay

Tube formation assays were conducted using a *μ*-slide angiogenesis kit (ibidi, Germany) according to the manufacturer's instructions. The *μ*-slide was coated with 10 *μ*l of undiluted Matrigel matrix, and 1 × 10^4^ cells were plated per well. After 5 h incubation, the network formation was observed by a microscope. The nodes, junctions, meshes, and total mesh areas were analyzed automatically using ImageJ software.

### 2.6. Cell Proliferation Assay

Cells were obtained during their growth phase, and 4 × 10^3^ cells were seeded into 96-well plates. Cell proliferation assays were then conducted using a Cell Counting Kit-8 (DOJINDO, Japan) according to the manufacturer's protocol. A well without cells served as a blank, and the absorbance of each well was measured at 450 nm using a spectrophotometer (TECAN, Mannedorf, Switzerland) at 24, 48, and 72 h after seeding. Finally, the cell viabilities of stably transfected cells were calculated using HTR8/SVneo cells as a reference (represented as 100%).

### 2.7. Cell Apoptosis Assay

Cells were harvested 48 h after seeding with 0.25% trypsin without EDTA (Solarbio, Beijing, China) and washed twice with 10 mM phosphate-buffered saline (PBS). The collected cells were then double-stained with annexin V-APC/7-AAD (KeyGEN BioTECH, China) according to the manufacturer's instructions. Fluorescence intensity analysis was performed using a flow cytometer (Cytomics FC 500, Beckman Coulter, USA) and CXP Cytometer analysis software (Beckman Coulter) to sort viable, necrotic, early apoptotic, and late apoptotic cells.

### 2.8. MAPK Pathway Phosphorylation Array and Western Blotting

Total phosphorylated cellular proteins were extracted with 1x cell lysis buffer containing protease inhibitors and two phosphatase inhibitors (Raybiotech, USA). The total protein concentration was determined using a BCA assay kit (Thermo Fisher Scientific, USA), and 17 phosphorylated MAPK pathway proteins were detected semiquantitatively using a Human and Mouse MAPK Pathway Phosphorylation Array C1 Kit (Raybiotech, USA) according to the manufacturer's protocol.

Rabbit monoclonal antibodies for phosphorylated p38 MAPK (pT180/pY182) and total p38 MAPK (Epitomics, USA) were used to detect the levels of phosphorylated and total p38 MAPK. Rabbit Phospho-RSK2 (Ser386), Western blot kit (Raybiotech, USA) and Rabbit monoclonal Anti-Rsk2 antibody (Abcam, UK) were obtained to measure the level of phosphorylated Rsk2 and total Rsk2. Total mTOR and phosphorylated mTOR (Ser2448) were detected by mTOR rabbit monoclonal antibody (Assay Biotech, USA) and Phospho-mTOR (Ser2448) polyclonal antibody (CST, USA). *β*-Actin was used as an internal control. Total proteins were separated using SDS-PAGE electrophoresis and then transferred onto PVDF membranes (Millipore, USA). Then membranes were incubated with primary antibody at 4°C overnight. Finally, an ECL substrate kit (Millipore, USA) was used for chemiluminescence detection. Gray values for each image were calculated using ImageJ software.

### 2.9. Pharmacological Inhibition Assay

The specific p38 MAPK inhibitors, SB203580 and BIRB796, were purchased from MedChemExpress (New Jersey, USA), and BMS582949 was purchased from Selleck (Houston, USA). Then three p38 MAPK inhibitors were dissolved in dimethyl sulphoxide (DMSO) for use. In order to choose the appropriate concentration of inhibitors, the three inhibitors were diluted from 0.1 *μ*M to 100 *μ*M for transwell invasion assay. Then the concentration which can correct the level of p-p38 of shRNA-HPSE-HTR8 to physiological level was chosen as the experimental concentration. Following pretreatment with 1 *μ*M BMS582949, 20 *μ*M SB203580, or 0.5 *μ*M BIRB796 for 2 h, shRNA-HPSE-HTR8 cells were collected for transwell invasion and tube formation assays. For the CCK8 assay, the absorbance was measured after the incubation of the cells with SB203580, BIRB796, or BMS582949 for 24 h, 48 h, and 72 h. After incubation with SB203580, BIRB796, or BMS582949 for 24 h, the rate of cell apoptosis was analyzed. Treatment with 0.1% DMSO was performed as a control.

### 2.10. Statistical Analysis

All continuous data coming from at least three independent experiments were expressed as the mean ± standard deviation (SD). Statistical analyses were performed using GraphPad Prism 5.02 (GraphPad Software Inc., San Diego, CA, USA). Statistically significant differences between the test and control groups were determined using two-tailed Student's *t*-test. Statistically significant differences among three or more groups were determined using one-way ANOVA. If the result of the ANOVA test was significant, the difference between the two groups was determined using Holm-Sidak's post hoc test. And *P* < 0.05 was considered to be statistically significant.

## 3. Results

### 3.1. Cell Line Authentication

The 22 STR loci of HTR8/SVneo cells were genotyped successfully. The STR profile of CSF1PO, D13S317, D16S539, D5S818, D7S820, TH01, vWA, TPOX, and amelogenin showed a 100% match between used HTR8/SVneo and the ATCC STR database profile (https://www.atcc.org/Products/All/CRL-3271.aspx#specifications). The electrophoretogram supporting cell line authentication is shown in Supplementary File 1.

### 3.2. Stably Transfected Cell Line Identification

Stably transfected HTR8/SVneo cells were constructed using an overexpression or a knockdown of the HPSE lentiviral vector. Expression of GFP was used as a marker of successful gene transfection (Supplemental Figures 1A–1E). The efficiency of transfection in HTR8/SVneo cells was evaluated using qRT-PCR (Supplementary Figure 1F). The expression of HPSE was markedly increased (~1000 fold) in HPSE-overexpressed cells (pLenti-HPSE-HTR8) compared with control cells (pLenti-HTR8) (*P* < 0.01). The expression of HPSE was decreased 2 fold in HPSE knockdown cells (shRNA-HPSE-HTR8) compared with control cells (shRNA-HTR8) (*P* < 0.05).

### 3.3. The Effect of HPSE on Trophoblast Cell Invasion

**Figure 1:**
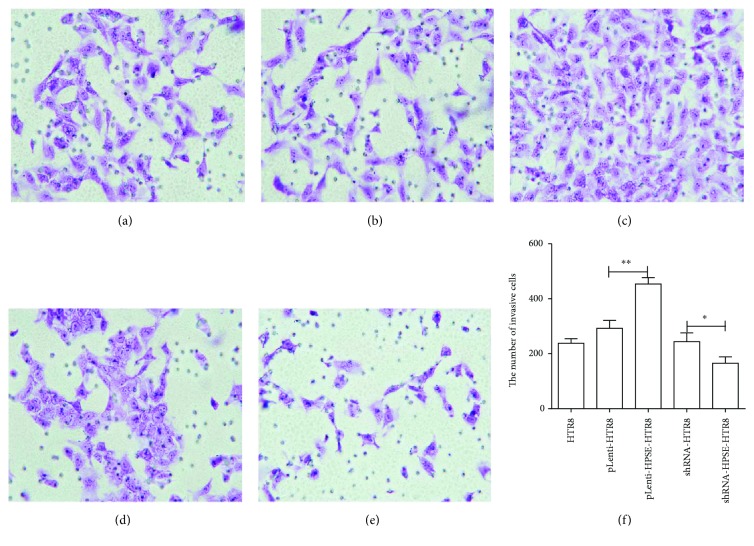
Effect of HPSE on trophoblast cell invasion. 5 × 10^4^ cells were suspended in 100 *μ*l of FBS-free RPMI-1640 medium and seeded into the upper chamber. 600 *μ*l RPMI-1640 medium with 10% FBS was added to the lower chamber. After incubation for 24 h, the invasive cells were fixed, dyed, and counted. (a–e) Representative images of transwell invasion assay. (a) HTR8, (b) pLenti-HTR8, (c) pLenti-HPSE-HTR8, (d) shRNA-HTR8, and (e) shRNA-HPSE-HTR8. Magnification: 400x (f). Quantification of the number of invasive cells. The number of invasive cells of fiver cell line was represented as the mean ± SD. The differences among HTR8, pLenti-HTR8, and shRNA-HTR8, between pLenti-HTR8 and pLenti-HPSE-HTR8, and between shRNA-HTR8 and shRNA-HPSE-HTR8 were compared by one-way ANOVA and Holm-Sidak's post hoc test. ^∗^ *P* < 0.05; ^∗∗^ *P* < 0.01.

The effect of HPSE on the invasion of HTR8/SVneo was assessed using a transwell invasion assay. The results indicated that invasion of pLenti-HPSE-HTR8 cells was markedly enhanced compared with pLenti-HTR8 cell. The number of invasive cells was 453.67 ± 23.25 in pLenti-HPSE-HTR8 cell but 292.33 ± 28.92 in pLenti-HTR8 cell (*P* < 0.01). In contrast, the knockdown of HPSE suppressed the invasion of HTR8/SVneo, and the number of invasive cells in shRNA-HPSE-HTR8 has decreased 1.5 folds than that in shRNA-HTR8 cell (*P* < 0.05) (Figures 1(a)–1(f)). The results indicated that HPSE could be a regulator for the invasion of EVTs.

### 3.4. The Effect of HPSE on Trophoblast Cell Tube Formation

**Figure 2:**
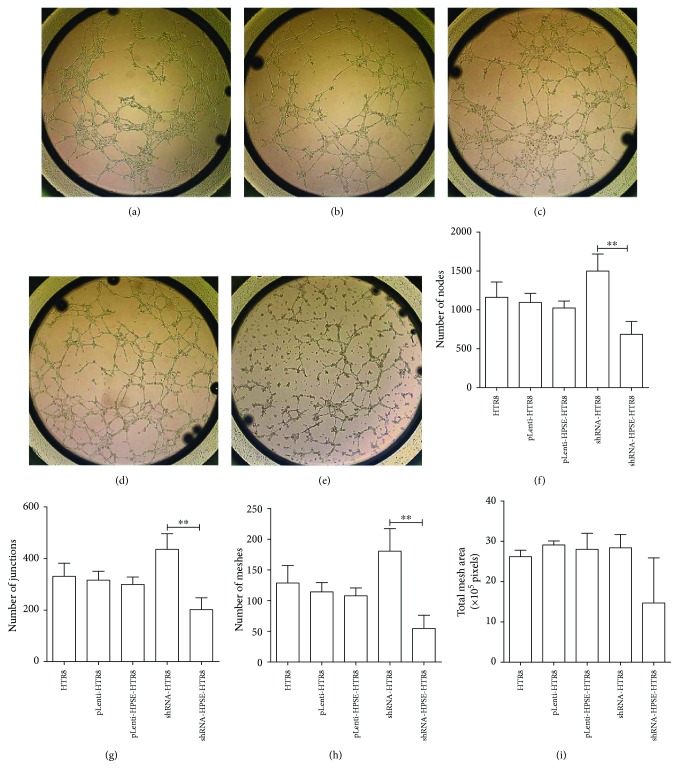
Effect of HPSE on trophoblast cell tube formation. 1 × 10^4^ cells were seeded on *μ*-slide angiogenesis plates, coated with 10 *μ*l of undiluted Matrigel matrix. After 5 h of incubation, the tube formation was observed and analyzed to count the number of nodes, junctions, meshes, and total mesh area by ImageJ software. (a–e) The tube formation on *μ*-slide angiogenesis plates. (a) HTR8, (b) pLenti-HTR8, (c) pLenti-HPSE-HTR8, (d) shRNA-HTR8, and (e) shRNA-HPSE-HTR8. Magnification: 40x. (f) Number of nodes. (g) Number of junctions. (h) Number of meshes. (i) Total mesh area calculated in pixels. All data were represented as the mean ± SD. The differences among HTR8, pLenti-HTR8, and shRNA-HTR8, between pLenti-HTR8 and pLenti-HPSE-HTR8, and between shRNA-HTR8 and shRNA-HPSE-HTR8 were compared by one-way ANOVA and Holm-Sidak's post hoc test. ^∗∗^ *P* < 0.01.

Previous studies have reported that HPSE promotes angiogenesis and lymphangiogenesis in tumor cells [6, 12]. To determine if HPSE expression has an influence on the proangiogenic properties of EVTs, tube formation assays were performed. As shown in Figures 2(a)–2(e), decreased tube formation was observed in shRNA-HPSE-HTR8 cells compared with control cells, while overexpression of HPSE had no significant effect on tube formation compared with control cells. The quantitative results demonstrated that the number of nodes and junctions was significantly reduced 2 folds by knockdown expression of HPSE, compared to the control group. Meanwhile, the meshes formed by shRNA-HPSE-HTR8 cells were 3 folds less than shRNA-HTR8 cells (*P* < 0.01) (Figures 2(f)–2(i)).

### 3.5. The Effect of HPSE on Trophoblast Cell Proliferation and Apoptosis

**Figure 3:**
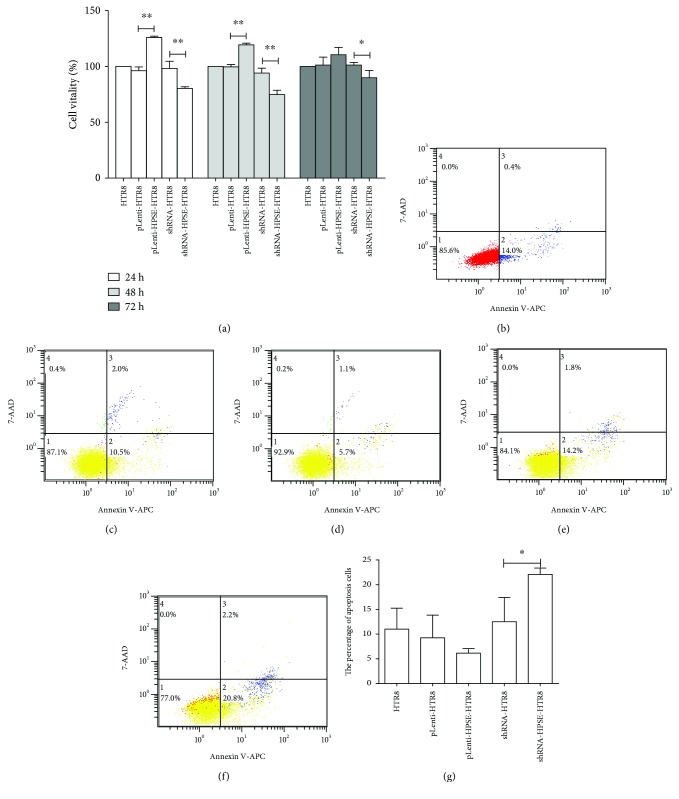
Effect of HPSE on trophoblast cell proliferation and apoptosis. (a) The rate of cell viability in 24 h, 48 h, and 72 h after seeding. HTR8 as a reference. Cells were conventionally cultured for 48 h, harvested with 0.25% trypsin without EDTA, and double-stained with annexin V-APC/7-AAD for flow analysis. (b–f) Flow cytometry analysis of cell apoptosis. (b) HTR8, (c) pLenti-HTR8, (d) pLenti-HPSE-HTR8, (e) shRNA-HTR8, and (f) shRNA-HPSE-HTR8. 1: viable cells. 2: early apoptotic cells. 3: late apoptotic cells. 4: necrotic cells. (g) The percentage of apoptosis cells in five cell lines. Data in graph a and graph g were represented as the mean ± SD. The differences among HTR8, pLenti-HTR8, and shRNA-HTR8, between pLenti-HTR8 and pLenti-HPSE-HTR8, and between shRNA-HTR8 and shRNA-HPSE-HTR8 were compared by one-way ANOVA and Holm-Sidak's post hoc test. ^∗^ *P* < 0.05; ^∗∗^ *P* < 0.01.

The CCK8 assay was conducted to examine the effect of HPSE on the proliferation of trophoblasts. Cell viabilities of pLenti-HPSE-HTR8 cells were 125.90% ± 1.20%, 119.33% ± 1.52%, and 110.54% ± 6.53%, and those of pLenti-HTR8 cells were 96.19% ± 3.34%, 99.58% ± 2.05%, and 101.25% ± 7.08% at 24, 48, and 72 h, respectively. Cell viability of pLenti-HPSE-HTR8 cells was significantly higher than that of pLenti-HTR8 cells in 24 h and 48 h (*P* < 0.01) but not in 72 h (*P* > 0.05). The viability of shRNA-HPSE-HTR8 cells was significantly lower than that of shRNA-HTR8 cells with 80.37% ± 1.36% versus 98.26% ± 6.32% in 24 h (*P* < 0.01), 74.79% ± 3.89% versus 94.09% ± 4.31% in 48 h (*P* < 0.01), and 89.88% ± 6.61% versus 101.31% ± 2.33% in 72 h (*P* < 0.05) (Figure 3(a)).

Harvested cells were double-stained with annexin V-APC/7-AAD and analyzed quantitatively by flow cytometry to investigate the apoptosis. The results indicated that the apoptosis of shRNA-HPSE-HTR8 cells was 1.8 folds higher than that of shRNA-HTR8 cells (22.10% ± 1.27% versus 12.50% ± 4.95%, *P* < 0.05). However, the rate of apoptosis in pLenti-HPSE-HTR8 cells was not significantly different from that of pLenti-HTR8 cells (*P* > 0.05) (Figures 3(b) and 3(g)).

### 3.6. Knockdown of HPSE Inhibited Trophoblast Cell Invasion by Activation of the p38 MAPK Pathway

**Figure 4:**
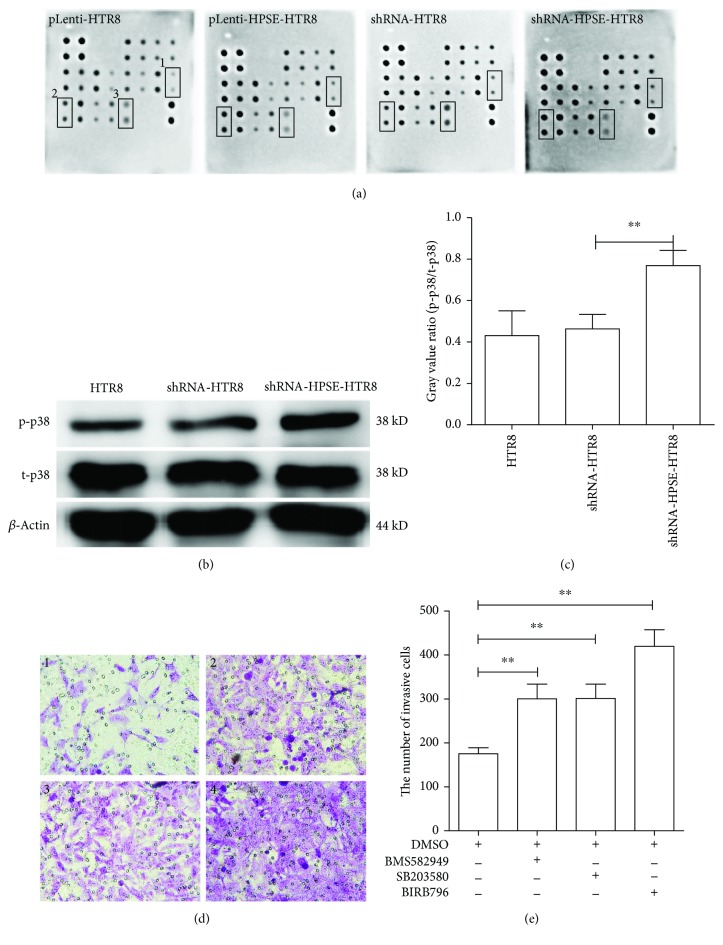
Knockdown of HPSE inhibited trophoblast cell invasion by activating p38 MAPK pathway. (a) Total cell protein including phosphorylated protein was extracted and analyzed by MAPK Pathway Phosphorylation Array C1. 1: p-mTOR. 2: p-p38 MAPK. 3: p-Rsk2. To confirm the results of microarrays, the three phosphorylated proteins and the corresponding total protein were determined in three independent experiments by Western blotting. (b) Western blotting images of p38 MAPK. (c) The gray value ratio of p-p38/t-p38 was elevated in HTR8/SVneo cells transfected with shRNA-HPSE. *β*-Actin was used as internal control. (d) Representative images of cell invasion after HPSE knockdown in shRNA-HPSE-HTR8 cells, pretreated with pretreated with 1 *μ*M BMS582949, 20 *μ*M SB203580, or 0.5 *μ*M BIRB796 for 2 h. 1: 0.1% DMSO as control. 2: 1 *μ*M BMS582949. 3: 20 *μ*M SB203580. 4: 0.5 *μ*M BIRB796. Magnification: 400x. (e) Quantitative analysis for the number of invasive cells. Data in graph c and graph e were represented as the mean ± SD. The differences among them were compared by one-way ANOVA and Holm-Sidak's post hoc test. ^∗∗^ *P* < 0.01.

To determine if the MAPK pathway was involved in mediating the role of HPSE in EVTs, the MAPK pathway phosphorylation was evaluated. The array results revealed that the level of p-p38 MAPK was increased (~2.47 folds) in shRNA-HPSE-HTR8 cells compared with shRNA-HTR8 cells and the level of p-Rsk2 was decreased approximately 1.8 folds in shRNA-HPSE-HTR8 cells compared with shRNA-HTR8 cells. The level of p-mTOR in pLenti-HPSE-HTR8 cells was elevated (~1.61 folds) compared to pLenti-HTR8 cells (Figure 4(a)). Furthermore, to validate the microarray data, these three phosphorylated proteins and the corresponding total protein were determined in three independent experiments by Western blotting. Semiquantitative analysis indicated that the ratio between phosphorylated p38 MAPK proteins and total p38 MAPK proteins was increased in shRNA-HPSE-HTR8 cells compared with shRNA-HTR8 cells (*P* < 0.01) (Figures 4(b) and 4(c)), while the other two had no significant differences (data not shown).

shRNA-HPSE-HTR8 cells were treated with three specific inhibitors of the p38 MAPK pathway (BMS582949, SB203580, and BIRB796) for further confirmation whether knockdown of HPSE in trophoblasts was associated with the activation of the p38 MAPK signaling pathway. The results indicated that SB203580, BIRB796, and BMS582949 had no obvious effect on shRNA-HPSE-HTR8 cell proliferation, apoptosis, and tube formation (data not shown). After pretreatment with 1 *μ*M BMS582949, 20 *μ*M SB203580, or 0.5 *μ*M BIRB697, transwell invasion assays revealed that the invasive ability of shRNA-HPSE-HTR8 cells was significantly restored compared with DMSO-pretreated shRNA-HPSE-HTR8 cells (*P* < 0.01) (Figures 4(d) and 4(e)). These results demonstrated that knockdown of HPSE inhibited HTR8/SVneo cell invasion by activating the p38 MAPK pathway.

## 4. Discussion

During the early stage of pregnancy, successful placental development, including EVT invasion and maternal spiral artery remodeling, is the most important factor for fetal development and gravida health, for which the adequate invasion of EVTs is a tightly controlled process and an important guarantee [13]. Inadequate EVT invasion and failure in spiral artery remodeling produce pregnancy-related disorders, including PE [14]. An understanding of the molecular mechanisms that are associated with regulating the invasiveness of EVTs is critical for explaining the cause and pathogenesis of PE. HPSE, the only known endogenous *β*-glucuronidase in mammals, has been shown to be positively connected with tumor cell invasion and metastasis [6]. Previously, we have demonstrated that HPSE expression and location in the placenta were significantly different between the severe PE and the normal groups [11]. Furthermore, Yang et al. demonstrated that HPSE in the nucleus played a tumor-suppressive role in melanoma progression [15]. In the present study, we demonstrated that HPSE expression had a positive influence on promoting the invasiveness of EVTs. EVT invasion was decreased by the downregulation of HPSE *in vitro*, which indicated that downregulation of HPSE might be a critical cause in the occurrence and development of PE from decreasing EVT invasion. However, a study by Harris et al. thought that trophoblast-derived heparanase was not required for invasion [16]. The cell lines they used were human first-trimester cytotrophoblast and the EVT-derived cell line SGHPL-4, but the cell lines we used were human first-trimester EVT cell line HTR8/SVneo. Maybe the differences in cell lines caused different results.

In addition, tube formation by HTR8/SVneo cells was inhibited by knockdown of HPSE, while there was no significant effect on tube formation when HPSE was overexpressed. When HPSE was overexpressed, HTR8/SVneo cells exhibited enhanced proliferation and invasion that was characteristically similar to choriocarcinoma (CCA). CCA is composed of neoplastic cytotrophoblasts, EVTs, and syncytiotrophoblasts, in which syncytiotrophoblasts, rather than EVTs, exhibit an endothelial-like function to form central pseudovascular channels as a blood supply for CCA growth and progression. However, placental site trophoblastic tumors and epithelioid trophoblastic tumors have true blood vessels that provide a blood supply inside the tumor, instead of the vasculogenic mimicry formed by EVTs. Thus, in trophoblastic tumors, syncytiotrophoblasts, but not EVTs, play an important role in tube formation [17]. This may be the reason that HPSE overexpression, which functionally induced trophoblasts to approach tumor cells, did not affect tube formation in HTR8/SVneo cells.

We also investigated the effect of HPSE on HTR8/SVneo cell growth and apoptosis. The results revealed that overexpression of HPSE promoted the proliferation of EVTs and downregulation of HPSE suppressed trophoblasts growth. We also found that inhibition of HPSE expression increased apoptosis of HTR8/SVneo cells, rarely reported in tumors. It has been reported that HS regulates the segregation and presentation of fibronectin (FN) [7, 18]. Other research has demonstrated that FN secretion could support cell proliferation in glioma [19] and knockdown of FN-limited tumor growth in ovarian cancer [20]. Furthermore, FN protects cells against apoptosis caused by docetaxel in lung cancer [21], and the degradation of FN could induce fibroblast apoptosis [22]. We therefore hypothesized that HPSE facilitates the proliferation of trophoblasts by modulating the secretion of FN by cleaving HS side chains and that knockdown of HPSE induces cell apoptosis by decreasing the secretion of FN.

The MAPK pathway has been reported to be involved in regulating the biological effect of HPSE in tumor cells such as nasopharyngeal carcinoma [23] and melanoma [24]. Thus, the mechanism by which HPSE controls the EVT invasion was thought to be relevant to the activation of the MAPK pathway. Our results demonstrated that p38 MAPK was upregulated by knockdown of HPSE, and when pretreated with BMS582949, SB203580, or BIRB796, the invasiveness of HTR8/SVneo cells with knockdown of HPSE was recovered. This revealed that the underlying mechanism by which knockdown of HPSE suppressed HTR8/SVneo cell invasion was by activating the p38 MAPK pathway, which was consistent with a previous report [25]. Proper phosphorylated p38 is necessary for the normal physiological function of the cells. He et al. have reported that 5 *μ*M BIRB796 obviously inhibited p-p38 in human oral epidermoid carcinoma cell line KB and KBV200 cell [26]. In our study, although 5 *μ*M BIRB796 could markedly decrease the level of phosphorylated p38 of shRNA-HPSE-HTR8 cell, it could not recover the invasion of this cell line, whereas 0.5 *μ*M BIRB796 which corrected phosphorylated p38 of shRNA-HPSE-HTR8 to the level of HTR8 could promote shRNA-HPSE-HTR8 invasion clearly. It suggested that the concentration of p38 MAPK inhibitors should be carefully considered when they were put into clinical practice in the future.

We also examined if p38 MAPK contributed to the effect of HPSE on EVT proliferation, apoptosis, and tube formation. However, none of them had any significant effect on proliferation, apoptosis, or tube formation in shRNA-HPSE-HTR8 cells. Since the potential mechanism for the effect of HPSE on EVT proliferation, apoptosis, and tube formation has not yet been explained, we plan to screen other potential pathways by transcriptome and proteomic analyses. This should help to elucidate the mechanism of action of HPSE.

## 5. Conclusion

In conclusion, our study is the first to reveal a strong association between HPSE and the behavior of EVTs. HPSE plays an important role in regulating the biological behavior of EVTs. Downregulation of HPSE, especially, may be a crucial factor in the pathogenesis of PE because of PE-like outcomes from decreased cell proliferation, invasion, and tube formation but increased apoptosis, in which the p38 MAPK pathway is involved. Thus, HPSE is a potential biomarker for predicting PE at an early stage of pregnancy and is a promising therapeutic target for PE.

## Supplementary Materials

Supplementary 1 File 1: electrophoretogram of HTR8/SVneo cell line authentication.

Supplementary 2 Figure 1: stably transfected cell line identification. (A–E) Schematic representation for GFP protein. (A) HTR8, (B) pLenti-HTR8, (C) pLenti-HPSE-HTR8, (D) shRNA-HTR8, and (E) shRNA-HPSE-HTR8. (F) Level of HPSE mRNA in five cell lines. ^∗^ *P* < 0.05; ^∗∗^ *P* < 0.01.
