# Supplementary figures and images for: Knockdown of Heparanase Suppresses Invasion of Human Trophoblasts by Activating p38 MAPK Signaling Pathway
Source: Dis Markers. 2018 Apr 17;2018:7413027. doi: 10.1155/2018/7413027 (PMC5932509; doi:10.1155/2018/7413027)

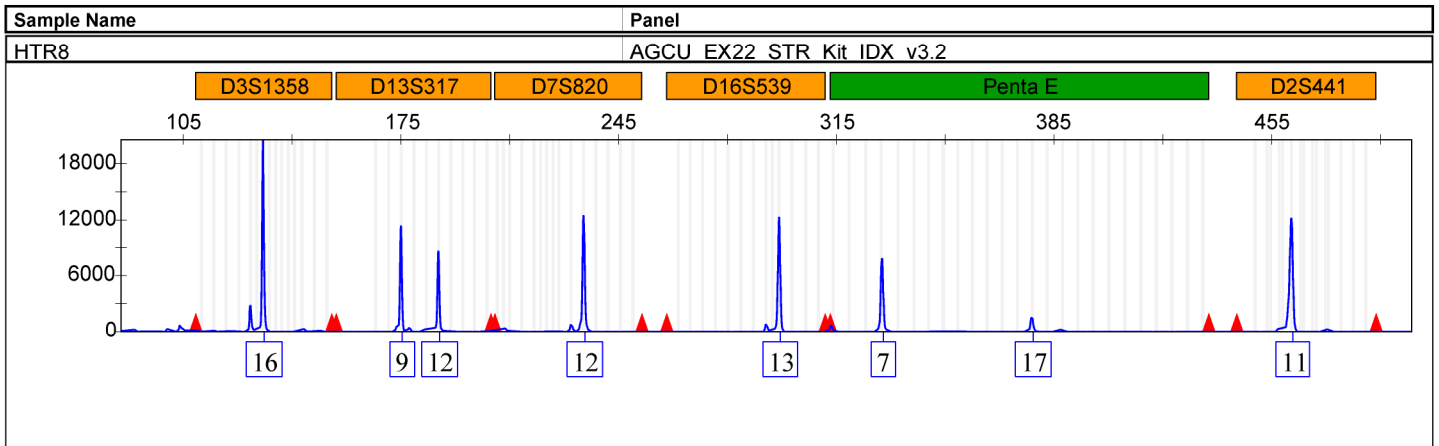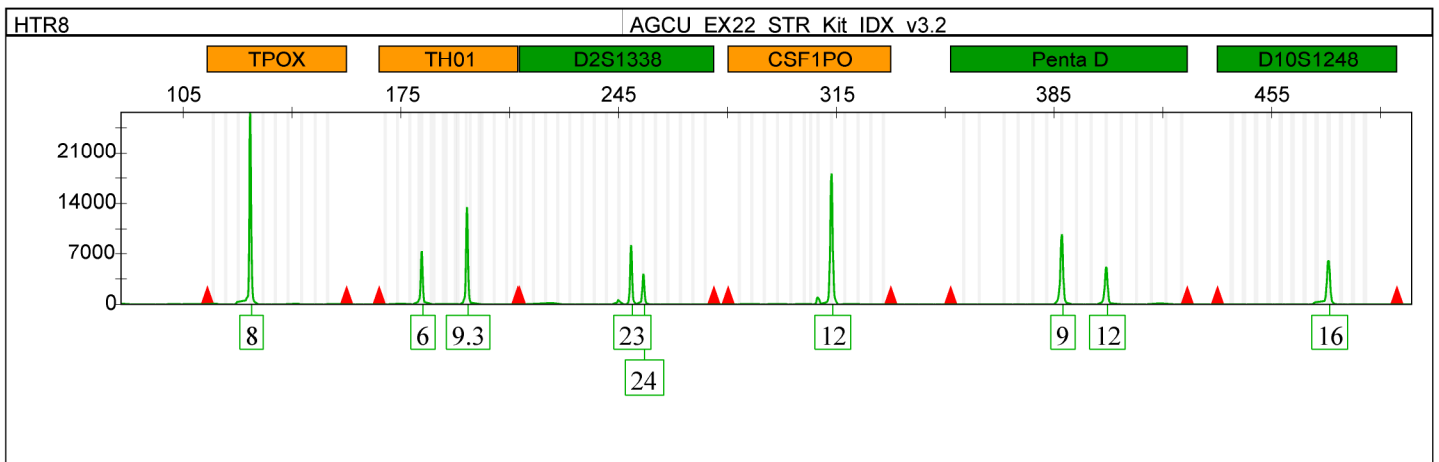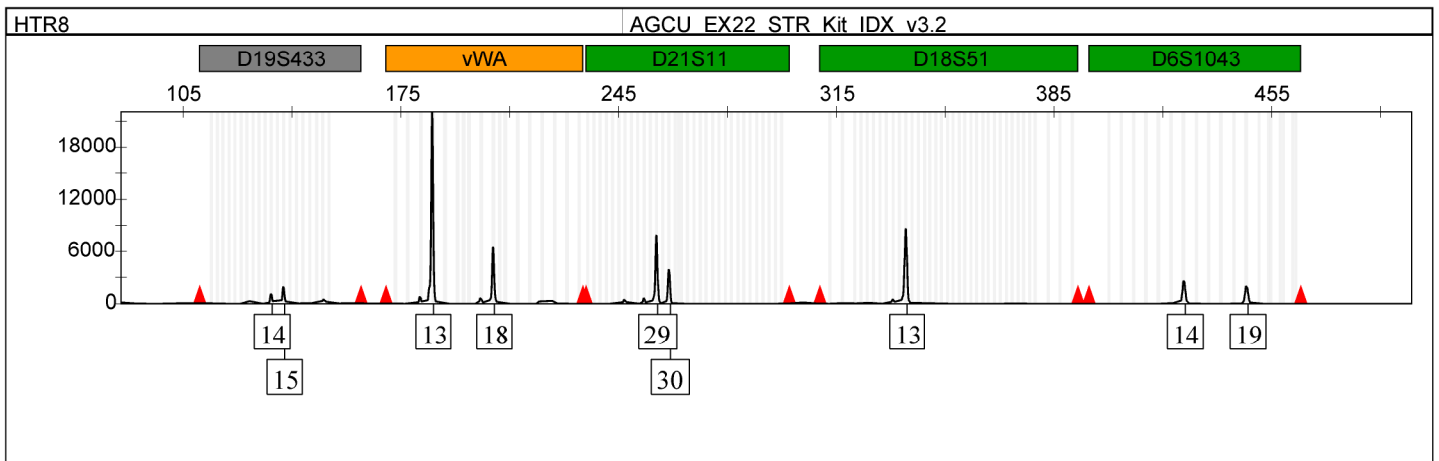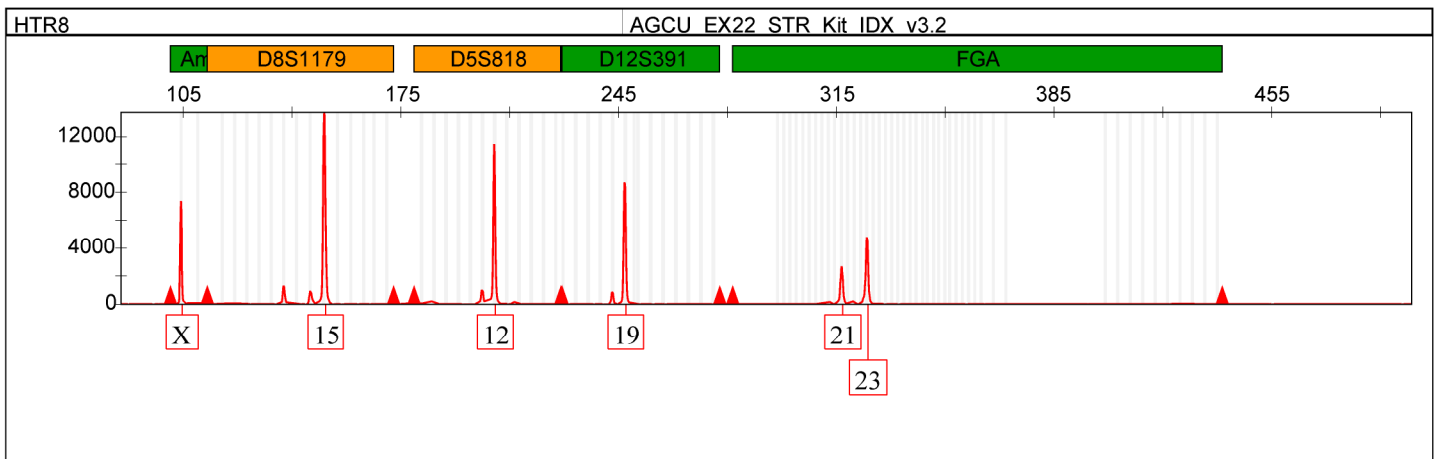

Supplement: Supplementary 1 — File 1: electrophoretogram of HTR8/SVneo cell line authentication. [file 7413027.f1.pdf]
